# Institutional Needs

**Published:** 1915-02-06

**Authors:** 


### Institutional Needs.

BOVININE.

(The Bovinine Co.—W. Edwards and Sons, Agents, 157 Victoria Street, London, E.C.)

We have received a specimen of Bovinine and most thoroughly tested it. It belongs to the class of substances known as meat juices. All meat juices are highly nutritive preparations containing varying quantities of coagulable proteid. Bovinine contains approximately 17 per cent. of such proteid, which, compared with other similar preparations, must be regarded as a distinctly high content. Bovinine can be given in teaspoonful doses diluted with water, milk, or weak wine and water, but, as is the case with all meat juices, it must not be heated or, at any rate, raised over blood heat. Bovinine also contains an organic iron compound, probably a hæmoglobin derivative, and hence some hæmatinic value can be reasonably ascribed to it. One attractive advantage Bovinine seems to possess over some of its competitors is its reasonable price. It is almost impossible to make a meat juice with a flavour which all consumers regard as appetising. A good taste can, however, be given by diluting Bovinine with any of the meat extracts, but always remembering that this dilution must take place in the cold. This preparation is to be recommended, and may be used with confidence.

